# Septic arthritis of knee joint after rooster attack: a case report

**DOI:** 10.1186/s13256-025-05215-0

**Published:** 2025-07-16

**Authors:** Florian C. Mackes, Oskar-Marek Kwaczynski, Michael T. Hirschmann, Natalie Mengis

**Affiliations:** Department of Orthopedic and Trauma Surgery, Kantonsspital Baselland, Standort Bruderholz, Gemeindeholzweg, 4101 Binningen, Switzerland

**Keywords:** Septic arthritis, Atypical bacteria, Animal injury, Penetrating wound, Arthroscopy

## Abstract

**Background:**

Septic arthritis is a serious orthopedic emergency that can lead to irreversible chondrolysis and joint destruction if not treated promptly. Although *Staphylococcus aureus* is the most common pathogen, atypical bacteria, especially injuries inflicted by animals, can cause severe septic arthritis. Recognizing the risk of infection from even seemingly minor injuries during initial inspection is crucial to prevent fulminant septic arthritis. Diagnostic tools such as blood cultures and synovial fluid aspiration are key to identifying the causative bacteria and guiding antibiotic therapy.

**Case report:**

A Swiss, 26 years old, woman got pecked by a rooster and suffered an injury to her right knee. The patient’s symptoms, blood analysis, and joint aspiration confirmed the diagnosis of septic arthritis of the knee. Empiric antibiotic treatment with amoxicillin and clavulanic acid was started. Arthroscopic debridement and dilution was initiated. The arthroscopic view showed damage to the right medial femur condyle, which was overlooked by the medical staff during the patient’s initial examination. The patient recovered quickly and showed no more restrictions 6 weeks after trauma.

**Conclusion:**

This case highlights the importance of recognizing the risk of infection, even from seemingly small injuries such as a rooster peck, to prevent fulminant septic arthritis. Further, this case demonstrates the importance of not underestimating a penetration wound by inspection only. Thorough clinical examination and wound exploration or saline load test can help to assess the depth of penetrating wounds. Nevertheless, rapid surgical and antibiotic treatment ensured a positive outcome for the patient in the case of this orthopedic emergency.

## Background

Septic arthritis (SA) is a serious orthopedic emergency that can lead to irreversible chondrolysis and joint destruction if not treated promptly [1]. In addition to previous joint infections, there are several risk and pathogenic factors that can increase the development of SA, such as diabetes, old age, or rheumatoid arthritis [2]. Although *Staphylococcus aureus* is the most common pathogen [3, 4], atypical bacteria such as *Enterococcus* can also cause SA, especially after injuries inflicted by animals [5–9]. Recognizing the risk of infection from even seemingly minor injuries is crucial to preventing fulminant SA [10]. Patients often present with signs of inflammation, including pain, swelling, redness, and fever [11–13]. Diagnostic tools such as blood cultures and synovial fluid aspiration are key to identifying the causative bacteria and guiding antibiotic therapy [10, 13]. Prompt and accurate treatment is essential to prevent serious complications [11].

This case report presents the clinical history of a young woman with SA following injury caused by a rooster’s beak, which resulted in damage to the right femoral condyle. This is the first reported case of a rooster attack leading to bacterial septic arthritis. Another case described in literature [14] showed prolonged healing owing to the inability to isolate the bacteria, highlighting the importance of early and effective intervention [14].

### Case presentation

A white Swiss woman, 26 years old, was admitted to the emergency department of our healthcare facility because of a painful, red, heated, and swollen right knee and a skin lesion on the inside of the knee. This injury was the result of an unfortunate incident involving a rooster attack the day before and caused immediate pain, swelling, and significant redness in the affected area. She immediately cleaned and disinfected the wound. The patient keeps chickens and roosters at home and was admitted to our emergency department 4 months earlier for a cat bite injury on her right hand. She had erysipelas and received antibiotic treatment with oral 875 mg amoxicillin and 125 mg clavulanic acid 3 times/day for 5 days. There was no prior history of gout, body rush, or knee swelling.

### Investigations

**Fig. 1:**
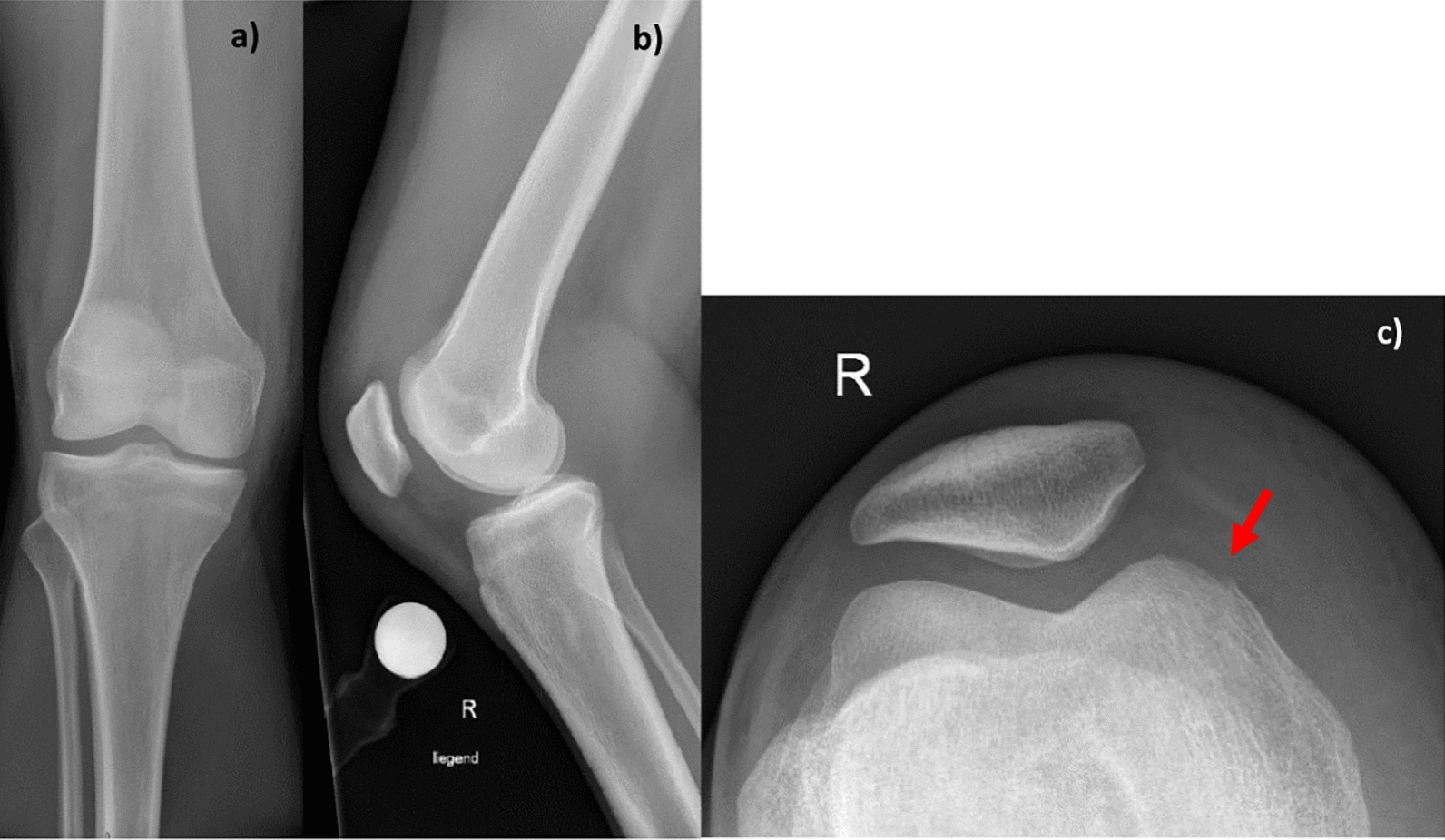
The radiographs of right injured knee taken at the initial appearance at our emergency department to see joint effusion and to rule out fracture. **a** Shows the anteroposterior view; **b** the lateral view; and **c** the tangential patella view. On image **c**, you can see the irregularity of the corticalis at the medial femur condyle (red arrow)

On clinical examination, the patient’s knee was swollen, red, warm to the touch, and displayed a 0.5 cm long skin lesion on the right medial femoral condyle. There was no discharge of pus, and no further evaluation of the depth of the lesion was made. The medical team decided to perform an intra-articular puncture to reduce the swelling, preserve any potential bacteria, and determine the nature of the joint effusion. Approximately 70 ml of fluid was aspirated during the procedure. The joint fluid was bloody, mucous in consistency, and visually cloudy. The joint fluid analysis revealed 3.5 mmol/l glucose, 41.3 g/l protein, and a remarkably high total cell count of 47,980 × 106/l, with the same amount of leukocytes at 47,980 × 106/l. In addition, 97.2% of the cells observed were identified as polymorphonuclear cells (PMN). In the corresponding blood samples, C-reactive protein (CRP) was 30 mg/l (norm < 5 mg/l) and leukocytes were 11 × 109/l (norm 4–10 × 109/l). Radiological imaging revealed a significant effusion within the knee joint but no structural damage (Fig. 1).

There was microbiological evidence of pan-susceptible *Enterococcus* *faecium* in the enrichment culture of the initial joint aspiration. The intraoperative biopsies were all negative. The histological results showed florid inflammation of the suprapatellar recessus. The individual bone and cartilage fragments of the medial knee joint showed florid inflammation, the intercondylar tissue a fibrinoleukocytic exudate, and minor florid inflammation at the lateral recessus.

### Differential diagnosis

The diagnosis was septic arthritis, which was most likely due to the penetrating wound, which exhibited subsequent redness, swelling, and overheating. In addition, the macroscopic appearance of the joint aspiration and the results of the aspiration were consistent with infection. A differential diagnosis of fracture can also present with swelling, hyperthermia, and increased inflammatory parameters.

There are several laboratory findings from the synovial fluid that can help differentiate between bacterial infection and crystal-induced arthritis. The macroscopic appearance of the synovial fluid can help differentiate septic arthritis from noninfectious conditions such as gout. In septic arthritis, the fluid is typically turbid, cloudy, and purulent owing to elevated leukocyte levels and the presence of bacteria, often appearing thick and yellow-green [15]. Conversely, in gout, the fluid is cloudy owing to the presence of crystals but is not purulent [16]. White blood cell counts (WBC) further differentiate these conditions. Septic arthritis is characterized by WBC counts above 50,000 cells/μl, with neutrophils making up more than 90% of the population [1]. Gout typically presents with lower WBC counts between 2000 and 50,000 cells/μl, with neutrophils still predominant but in smaller proportions [17]. Polymorphonuclear neutrophils are elevated in both conditions, but in septic arthritis they represent more than 75% of the WBC count, reflecting a robust neutrophilic response [1]. In gout, PMNs represent 50–70% of the total WBC count [15]. Crystal analysis is crucial in the diagnosis of gout, with monosodium urate crystals (negatively birefringent) confirming gout and calcium pyrophosphate crystals (positively birefringent) being seen in pseudogout [15]. No crystals are seen in bacterial infections. Finally, synovial fluid glucose concentration is significantly lower in septic arthritis (< 50% of serum glucose), suggesting bacterial glucose consumption [17]. In gout, glucose levels are typically normal or only slightly decreased, emphasizing the noninfectious etiology [16].

### Treatment

**Fig. 2:**
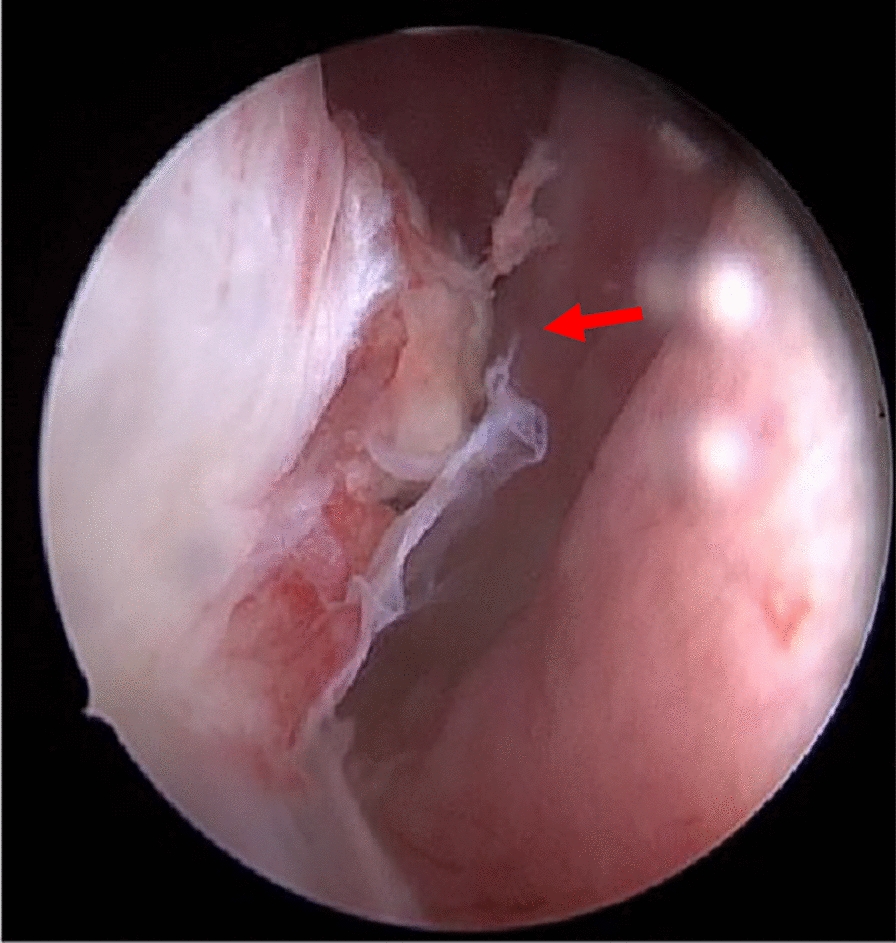
The image shows an intra-arthroscopic view at the border of the medial femoral condyle. The red arrow points to the fracture at right medial femur condyle injury before debridement

**Fig. 3:**
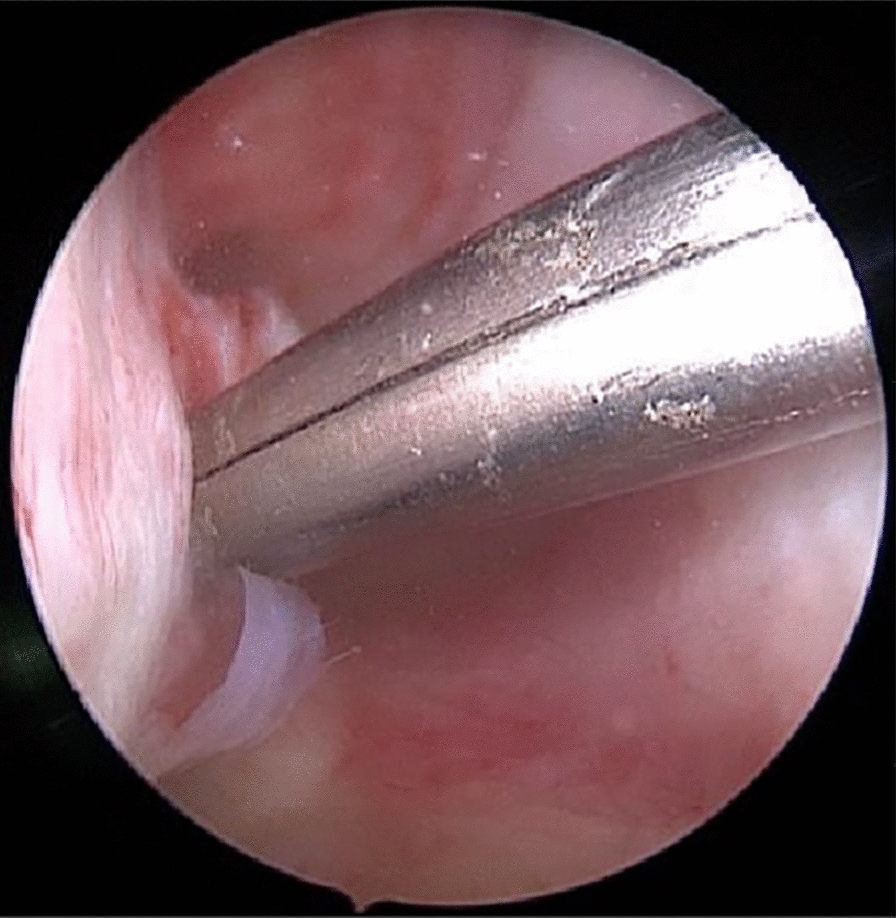
The intra-arthroscopic image shows the correlation of the external skin lesion and medial femur condyle injury with a straight metal rod

**Fig. 4:**
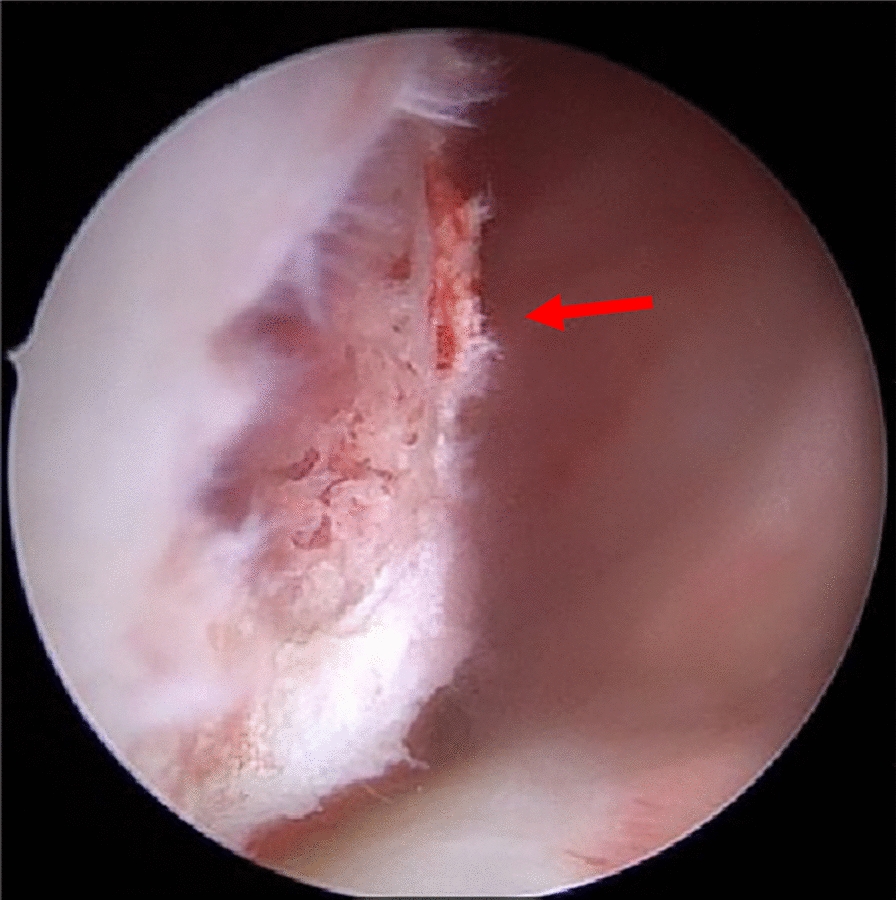
In this intra-arthoscopic picture, the red arrow points to a bony spongiosa after debridement of the lesion at the right medial condyle

The medical team recommended immediate knee arthroscopy, joint irrigation, and biopsy for further diagnostic clarification and thorough wound revision. In accordance with best practice guidelines, the patient provided informed written consent for the proposed procedure. Knee arthroscopy was performed around 8 h after appearance at the emergency department by a resident and a consultant using a working pressure of 80 mmHg. According to current guidelines [13], the joint was diluted with 9 l of Ringer’s solution, debrided, the chondral lesion was smoothed with a shaver, and a free joint body was removed. Arthroscopy revealed purulent material, severe inflammation, and fibrinous deposition, consistent with stage II arthritis according to the Gächter classification [18]. In addition, there was a bony-cartilaginous lesion on the medial aspect of the right medial femoral condyle (Fig. 2). During arthroscopy, the solution spurted through the skin tear, confirming an intracapsular lesion due to the rooster’s beak (Figs. 3 and 4). After arthroscopy, the edges of the skin laceration on the right medial knee were debrided, and the wound was washed and fitted with Ethilon 3.0 single button sutures. Empiric antibiotic therapy with 2000 mg amoxicillin and 200 mg clavulanic acid intravenous 3 times/day was started immediately intraoperatively after sampling. Postoperatively, CRP decreased to 23 mg/dl. The clinical symptoms (redness, swelling, and pain) improved, and the patient was able to leave the hospital 2 days after surgery. Antibiotic therapy was continued with oral 875 mg amoxicillin and 125 mg clavulanic acid 3 times/day for another 9 days.

### Outcome and follow-up

**Fig. 5:**
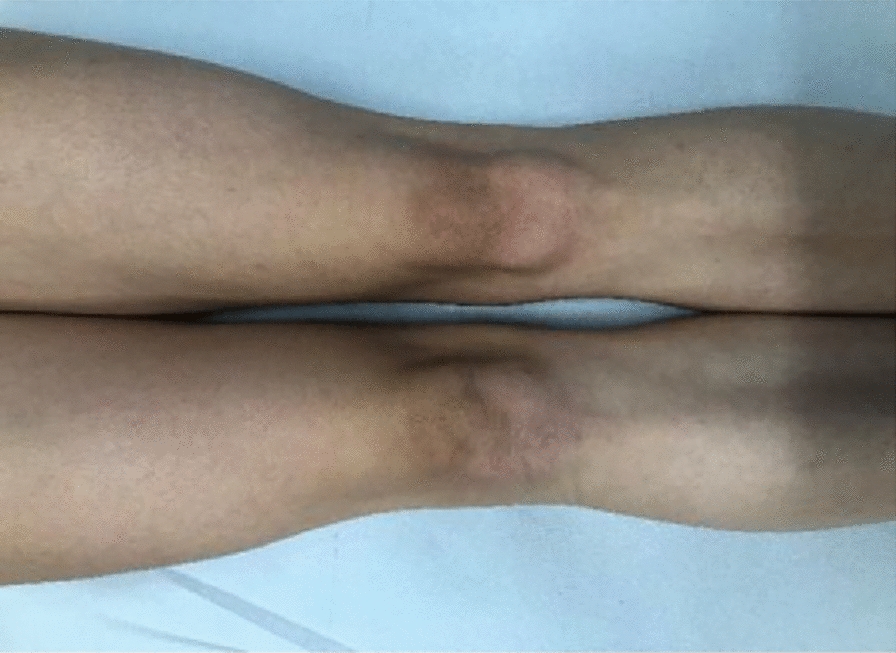
This photograph was taken 3 months after trauma. The comparison of both knees on an anterior aspect demonstrates irritation-free scars and no signs of swelling in the right knee

Subsequently, 2 weeks after surgery, the sutures were removed, and the patient had no further complaints. There was a positive clinical outcome 2 months after the accident. The patient was able to perform a deep squat completely and without pain. There was no sensory or motor deficit, no significant intra-articular effusion, and no tenderness around the arthroscopy portals, which were soft and did not perturb the subcutaneous layer. There was no swelling, redness, or hyperthermia around the knee joint on examination (Fig. 5). There was still minimal discomfort to palpation around the medial femoral condyle. Functionally, the patient had a ligamentous stable knee joint with no pain on functional testing of the menisci or patella. She has not complained of any knee problems 10 months after the accident.

## Discussion

### Epidemiology, risk factors, and pathogenesis

The diagnosis of SA is based on the modified criteria of Newman [19]. The criteria are the following: isolation of a pathogen from an affected joint; isolation of a pathogen from another source (e.g., blood) in the context of a hot red joint suspicious for sepsis; typical clinical features and cloudy joint fluid in the presence of previous antibiotic treatment; and postmortem or pathological features suspicious for septic arthritis.

Rarely, the diagnosis cannot be established microbiologically, but is highly suspected clinically, and antibiotic therapy is started before synovial fluid aspiration.

Accurate epidemiological data collection in SA is challenging owing to the retrospective nature of data collection. The incidence of proven or probable septic arthritis in Western Europe is 4–10 per 100,000 patient-years per year [3, 10, 11]. It appears to be increasing, mainly owing to an aging population, more invasive procedures, and increased use of immunosuppressive treatment [12]. The risk of SA is approximately four per 10,000 corticosteroid injections [12]. Post-arthroscopic septic arthritis has a prevalence of approximately 14 per 10,000 procedures (0–14%) [20]. Several case reports of SA following penetrating animal injuries have been described in literature. There are common causes of septic arthritis following animal attacks, more commonly associated with cat, dog, or rat bite injuries [5, 6]. There are rare cases of septic arthritis caused by a catfish bite [7], a rattlesnake bite [8], or a seal bite [9]. Potential risk factors of SA are rheumatoid arthritis or osteoarthritis, previous implantation of a joint prosthesis, low socioeconomic status, intravenous drug abuse, alcoholism, diabetes, previous intra-articular corticosteroid injection, and cutaneous ulcers [2]. For younger people, skin infections [3], joint injections with corticosteroids, and penetrating injuries are risk factors for SA [20, 21].

*Staphylococcus aureus* is the most frequently identified causative bacteria for SA followed by other gram-positive bacteria, including streptococci [3, 11, 22]. In addition to direct inoculation, infection can reach the joint by hematogenous spread. Bacteremia leading to SA is more likely to occur in older immunosuppressed patients with intravascular devices or urinary catheters [3]. This is consistent with literature describing patients aged 80 years and older with an increased prevalence of gram-negative organisms, presumably owing to a higher prevalence of comorbidities, including urinary tract infections and skin ulcers [23]. Intravenous drug users have an increased susceptibility to mixed bacterial and fungal infections and unusual organisms [3, 11, 22].

### Clinic and diagnostic

Patients with septic arthritis typically present with a short history of redness, pain, limited range of motion, and restricted joint mobility [11–13]. Some factors, including low virulence of the causative organisms and fungal or mycobacterial infection, can delay presentation [24]. In this case, the injury to the capsule of the knee joint appears to have led to the entry of a large quantity of bacteria, in this case *Enterococcus* *faecalis*. Together with the cortical injury, this could explain the patient’s rapid and pronounced symptoms.

According to Kongmalai’s systemic review in 2017, saline loading is an efficient option to detect or rule out capsular injury of the knee after penetrating wounds [25].

Systemic symptoms, such as fever or sweating, are less common than one might expect [24, 26], indicating that (in contrary to popular medical opinion) raised temperature is not a prerequisite for diagnosis of septic arthritis.

Blood cultures should always be taken before starting antibiotic treatment to increase the chances of obtaining the causative organisms [13]. The erythrocyte sedimentation rate, C-reactive protein concentration, and white blood cell count are usually elevated in blood samples from patients with septic arthritis. However, normal values for these variables have been reported at presentation, so the absence of an acute phase response does not exclude septic arthritis [26–28]. Nevertheless, these laboratory parameters should be measured since, if elevated, they are useful in monitoring response to treatment. In addition, renal and hepatic function should be assessed at presentation, as abnormalities may affect the choice or dosage of antibiotics, and impaired renal and hepatic function are poor prognostic factors in septic arthritis [29]. Urgent aspiration of joint fluid is an essential step in the management of suspected SA [30]. In cases where an endoprosthesis is present, aspiration of synovial fluid should be performed in an operating theater under full aseptic precautions. Gram stain, microbiological cultures and an antibiogram are the most important factors in the choice of antibiotic treatment. In the study by Hughes *et al*. [31], gram staining of synovial fluid identified the causative organism in 50% of cases, rising to 67% after culture [32]. To diagnose crystal arthritis, samples of synovial fluid should be examined by polarizing microscopy [33]. Whether quantification of the synovial leukocyte count is helpful in the diagnosis is controversial. Some investigators have suggested that this variable is a useful discriminator of septic arthritis, citing a threshold of more than 50,000 cells per μl or more than 90% polymorphnuclear cells [34–36]. Others have reported that this measure cannot distinguish between crystal arthritis and SA [37–39].

### Treatment and prognosis

The Guideline for Management of Septic Arthritis in Native Joints (SANJO) by Ravn *et al*. [13] can help clinicians in the management of patients with septic arthritis in native joints. The patient in the report was treated according to this guideline. As rooster beak injuries are not an everyday injury, there are no current guidelines for empirical antibiotic therapy. Given the potential pathogens of aerobic and anaerobic bacteria (*Staphylococci*, *Streptococci*, *Corynebacterium*, *E.* *coli*, and *Enterococcus* *faecium*), empirical antibiotic therapy with amoxicillin and clavulanic acid would be the treatment of choice [40, 41].

Post SA, poor functional outcome was recorded in 24% of individuals and osteomyelitis in a further 8% [18]. In children, residual symptoms after SA are common (25%) at long-term follow-up [42]. In case of implant-associated infection, consensus literature recommends urgent debridement, lavage, implant removal, and antibiotic therapy [13].

Comparing our case with the only other case of a chicken attack described in literature, some differences can be identified. In the case of Huang *et al*. [14], a 9-year-old girl was bitten on the lateral knee by a chicken. The initial presentation at the hospital occurred 2 weeks after the incident. Initial antibiotic therapy with oxacillin 6 g/day and ceftriaxone 2 g/day and daily punctures were performed. On day 9 of hospitalization, a knee arthroscopy with irrigation was performed owing to persistent pain and swelling. Subsequently, 2 months postoperatively, the patient was still complaining of pain and restricted knee movement. In comparison to this case from 1998, our case shows a better outcome 2 months postoperatively. This may be owing to the immediate and aggressive treatment regime. In addition, the arthroscopic technique nowadays has improved compared with the one used in 1998. An arthroscopic pressure of 80 mmHg was used. A higher intra-articular pressure results in a higher flow, which enables better irrigation of the joint and dilution of bacteria. However, a higher intra-articular pressure may push the bacteria into the surrounding tissue. Whether a lower arthroscopic pressure with only gravity inflow shows a better functional outcome has not yet been demonstrated [43, 44]. The creation of a third arthroscopic portal does not improve the effectiveness of arthroscopic lavage [45].

Injury to the femoral condyle impressively illustrates the underestimation of the depth of penetrating wounds, if they are only assessed externally [46, 47]. A lesion of the synovial membrane increases the risk of intra-articular entry of bacteria and, thus, septic arthritis.

The low rate of bacteria identification of causative organism [31] could be explained by dilution or flushing of the bacteria due to the arthroscopic procedure.

The microbiological analysis of the joint fluid aspiration revealed *Enterococcus* *faecium* in the enrichment culture of the initial sample. Roosters are frequently in contact with a wide variety of environmental microorganisms. On farms or in fields, it is plausible that the rooster’s beak became contaminated. This could conclusively explain the presence of *Enterococcus* *faecium* in the joint fluid. These findings highlight the importance of considering environmental contamination and atypical pathogens in cases of animal-related injuries.

A clinical follow-up of only 2 months is too short to evaluate the long-term outcome of the patient. To assess the long-term functional outcome and the cartilage damage, a follow-up of at least 1–15 years is necessary to evaluate the residual cartilage defect [48].

## Conclusion

This patient’s case is a powerful reminder not to underestimate penetrating wounds by only external inspection. A thorough clinical exploration and examination are crucial in penetrating wounds. Saline loading test can help to detect or rule out knee capsule injuries.

Further, this case of SA, as an orthopedic emergency, illustrates the importance of prompt medical care with initiation of antibiotic therapy after joint fluid aspiration. In adhering to provided guidelines, the early, effective, and compassionate surgical debridement and arthroscopic dilution for our patient resulted in a good short-term outcome. The long-term effect of this event with cartilage damage will be seen in the future.

## Data Availability

No underlying data are available for this article, since no datasets were generated or analyzed during this study.

## Abbreviations

SA: Septic arthritis
E. coli: *Escherichia* *coli*
WBC: White blood cell
CRP: C-reactive protein
PMNs: Polymorphonuclear neutrophils
